# Resilience and Emotional Intelligence of Staff Nurses during the COVID-19 Pandemic

**DOI:** 10.3390/healthcare10112120

**Published:** 2022-10-22

**Authors:** Bader Emad Aljarboa, Eddieson Pasay An, Wireen Leila Tanggawohn Dator, Salman Amish Alshammari, Romeo Mostoles Jr., Ma Mirasol Uy, Nojoud Alrashidi, Maha Sanat Alreshidi, Enrique Mina, Analita Gonzales

**Affiliations:** 1 King Khalid Hospital, Ministry of Health, Hail 55421, Saudi Arabia; 2 College of Nursing, University of Hail, Hail 81491, Saudi Arabia; 3 Department of Medical-Surgical Nursing, College of Nursing, Princess Nourah Bint Abdulrahman University, Riyadh 11671, Saudi Arabia; 4 Faculty, Philippine Public Safety College, Quezon City 1105, Philippines; 5 Department of Nursing, Faculty of Applied Medical Sciences, University of Tabuk, Tabuk 71491, Saudi Arabia

**Keywords:** COVID-19, resilience, emotional intelligence, nurses, workplace

## Abstract

Although numerous scholars have studied resilience during the COVID-19 pandemic, research exploring its relationship with emotional intelligence is scarce. The aim of this study was to determine the relationship between the resilience and emotional intelligence (EI) of staff nurses during the COVID-19 pandemic. Data for this quantitative correlational study were gathered from the staff nurses of hospitals in the city of Hail, Saudi Arabia. The researchers employed simple random sampling, which yielded 261 staff nurses. Nationality (*t* = 6.422; *p* < 0.001) was found to have a significant relationship with resilience. Sex (*t* = 5.22; *p* < 0.001), ward assignment (*t* = 5.22; *p* < 0.001), age (*F* = 6.67; *p* < 0.001), and years of experience (*F* = 6.67; *p* < 0.001) revealed significant relationships with emotional intelligence. Resilience had a moderate positive relationship with EI (*r* = 0.55; *p* < 0.023), a weak positive relationship with self-emotion (*r* = 0.21; *p* < 0.003), and a very strong relationship with emotional appraisal (*r* = 0.85; *p* < 0.001). Improving emotional-intelligence skills is critical for assisting nurses during pandemic outbreaks. This can increase their individual and social resilience, while also improving their professional and life outcomes. These research findings suggest that emotional intelligence should be integrated into clinical practice and that EI data should be integrated into decision-making.

## 1. Introduction

The COVID-19 pandemic has had a substantial influence on emotional and psychological diseases among healthcare workers, particularly nurses [1], as they have been caught in multifaceted ethical quandaries [2]. Threats related to contagion, physical fatigue, psychological despair, health problems, a lack of understanding, and interpersonal ambiguity have resulted in adverse emotions, including fear, worry, and helplessness [3]. Although nurses have been required to provide more reassuring care, they have also had to leave patients unattended to attend to those who are more vulnerable [4] due to an influx of patients in hospitals and the resulting staff shortages. Thus, nurses experience work-related stress as a result of the strain of having to carry out their responsibilities in difficult circumstances. Such situations force them to put their values and beliefs aside, causing frustration and disappointment. As the number of verified cases continued to rise, nurses confronted high-stakes decision-making difficulties that influenced both their work and personal development [5]. Cultivating emotional resilience allows nurses to perform their tasks while remaining emotionally separated from their patients and focused on the beneficial aspects of patient care rather than the unpleasant ones [6]. It helps them learn to overcome these challenges [7] while coping with environmental demands and pressures through emotional control [6]. 

Emotional intelligence (EI) has been defined as a person’s set of abilities and talents related to their psychological, emotional, and social lives that enables them to deal with pressure and demands from their surroundings [8]. EI involves the capability to be attentive to one’s own feelings as well as those of others, to be in control of one’s own actions, to encourage and influence others, and to regulate emotions effectively. It can be fostered to promote emotional, intellectual, and professional progress [6]. As such, nurses must be sufficiently robust to respond in ways that minimize their distress, while intelligently maintaining their emotions and resilience. 

Resilience is the mental and behavioral process of promoting personal resources and protecting oneself from the detrimental impacts of adversity [9]. This definition implies that mental toughness may act as a protective factor in the resilience process. The importance of properly managing one’s own immediate environment and developing protective and promoter variables that can be employed to build resilience can be understood in this context. When an individual views resilience as a beneficial trait rather than a stress reaction, the result is positive adaptation [10]. Resilience has been put to the test for many during the COVID-19 pandemic. Many nurses, for example, have had to decide how to allocate minimal resources [11] while attempting to deliver high-quality care in hazardous conditions [12], and they have been unable to deliver the highest level of care due to the large number of patients that they had to serve [13]. Such challenging situations expose healthcare workers to potentially harmful consequences (including stress). 

Although numerous scholars have studied resilience during the COVID-19 pandemic [14,15,16,17,18], research exploring its relationship with emotional intelligence is scarce. An earlier study revealed that resilience and emotional intelligence play a significant role in social support [13]. Other studies have found a positive association between them [11,12], and EI has been considered an antecedent to resilience [19]. Di Fabio and Saklofske [20] found the link between EI and resilience to be solid and complex. Some researchers have concluded that resilience and EI have a weak but positive relationship [21]. Nursing research in the setting of a pandemic is limited with regard to resilience and emotional intelligence. Research has linked the pandemic to elevated levels of depression, anxiety, stress, and burnout among healthcare personnel who provide direct care for COVID-19 patients, particularly frontline nurses [22,23]. In light of this research, it is expected that nurses’ resilience is affected by their ability to recognize and control their emotions. Understanding the context of resilience and emotional intelligence during the pandemic can teach nurses how to articulate their mental well-being and emotions so that they can learn about the abilities that help them build relationships and become more resilient.

To gain an understanding of and explain resilience and EI, the present research employed a descriptive correlational study to investigate the relationship between them in the context of the COVID-19 pandemic, which policymakers can use to study the results that can contribute to preparing nurses to think critically and integrate resilience and emotional intelligence into clinical practice. Doing so will allow them to improve both their nursing performance and their patient outcomes, and this will add to the body of knowledge on the subject. This study aimed to determine the relationships between the demographic characteristics of nurses and their resilience and emotional intelligence during the COVID-19 pandemic, as well as the relationship between resilience and emotional intelligence.

## 2. Materials and Methods

### 2.1. Design

A descriptive correlational research approach was employed to investigate the relationship between resilience and emotional intelligence.

### 2.2. Participants

The study participants were staff nurses employed by government hospitals in the Hail region of the Kingdom of Saudi Arabia, specifically Hail General Hospital, King Khalid Hospital, and King Salman Specialist Hospital. The Raosoft online calculator (http://www.raosoft.com/samplesize.html) (accessed on 4 December 2021) was used to compute the sample size with a 95% confidence interval, indicating that 261 participants were required. The researchers employed a simple random sampling technique and a number generator to choose the 261 participants from the 806 nurses who were listed as eligible to participate. A unique sequential number was assigned to each participant listed by the Ministry of Health. The numbers assigned to each eligible participant were derived from a list of random numbers generated from an automatic random number generator program. Inclusion criteria were nurses who had provided direct care to patients, whether on the COVID or non-COVID ward, had more than one year of working experience in the Kingdom of Saudi Arabia before COVID-19, who understood English (based on self-evaluation), and who were willing to participate in the study.

### 2.3. Data Collection

The researchers utilized a Google Form survey questionnaire, the link for which was forwarded through WhatsApp to the invited staff nurses. The researchers, together with key continuing nursing-education personnel, helped send the link to the participants. Through the link, the participants were provided with information about the study (e.g., the study objectives, extent of participation, and rights of participants). They were also provided with a note that instructed them to press the “open the link” button and respond to the questionnaire if they opted to participate. This study was conducted between October and November 2021. 

### 2.4. Questionnaires

The researchers used the original English versions of the questionnaires for data collection. There were three parts to the questionnaire, the first part being the demographic profiles of the participants (i.e., age, sex, and years of experience).

#### 2.4.1. Connor–Davidson Resilience Scale

The Connor–Davidson resilience scale was the second part of the questionnaire. It contained 10 items adapted from Campbell-Sills and Stein [24], which were on a cumulative 5-point Likert scale (0 = never to 4 = almost always). In all prior research carried out in industrialized countries, the 10-item Connor–Davidson Resilience Scale (CD-RISC) has shown excellent psychometric qualities as a measure of resilience [25]. The respondents answered based on how much they believed each item on the scale applied to them in the month prior to the study. The sum of the responses to each item provided a score from 0 to 40, with 40 indicating the highest level of resilience. Cronbach’s α was calculated by using SPSS version 26, and a score of 0.6 to 0.7 was considered acceptable. In this study, the resilience scale had an internal reliability of 0.78 based on the responses of 20 nurses.

#### 2.4.2. Emotional Intelligence Questionnaire

The third part was the emotional-intelligence questionnaire derived from Law, Wong, and Song [26]. It had 16 items across four dimensions: self-emotions, others’ emotions, use of emotion, and emotional regulation. In an earlier psychometric study conducted in Korea on EI, the final scale had a content validity index of 0.90, adequate construct validity, and an overall Cronbach’s alpha of 0.91 [27]. The nurses were asked to respond on a 7-point scale, with 1 indicating extreme disagreement and 7 indicating strong agreement. The internal reliability of the total emotional intelligence questionnaire in this current study was 0.81, using Cronbach’s α, which indicates excellent internal consistency. A pilot study of the two questionnaires was conducted before the actual study to predict the response patterns of the participants.

## 3. Ethical Considerations

This study was conducted with approval and clearance from the Ministry of Health, IRB #2021-51, dated 15 September 2021. 

## 4. Data Analysis

The data were analyzed by using SPSS version 26. The demographic parameters of the participants were determined by using frequency and percentage. The Kolmogorov–Smirnov test was used to determine the distribution of the data under the presumption that they were normally distributed. The Kolmogorov–Smirnov test result was high (0.93; *p* = 0.05), indicating that the data were normally distributed. Therefore, a one-way analysis of variance (ANOVA) and a *t*-test were used to examine the differences in participant demographics, resilience, and emotional intelligence. To estimate the strength of the links in the population that the data were sampled from, the researchers ensured that the assumptions of Pearson’s correlations were met. According to Schober and colleagues [28], the four assumptions are that the data were obtained from a representative or at least random sample; both variables are continuous random variables with joint normal distribution; there are no relevant outliers; and each pair of x–y values is measured independently from all other pairs. Therefore, Pearson’s correlation coefficient was used to examine the association between resilience and emotional intelligence (bivariate *r*). 

## 5. Results

Table 1 presents the demographic characteristics of the staff nurses. Most of the staff nurses in the workforce were 31–35 years old (34.1%), followed by 26–30 years old (29.9%). More female (53.6%) than male nurses (46.4%) participated. A total of 35.2% had 6–10 years of experience, and 31.0% had 5 years of experience or less. More than half of the nurses were designated to the COVID ward (57.9%) during the pandemic. The majority (54.1%) of the participants was expatriate nurses. 

Table 2 presents the differences between demographic characteristics, resilience, and emotional intelligence. The participants showed no significant difference in resilience according to sex (*t* = −1.013; *p* = 0.886). However, emotional intelligence was found to be significantly higher in female nurses (*t* = −7890; *p* < 0.001) than in male nurses (5.78 ± 0.644). 

There was no significant difference in resilience (*t* = 0.456; *p* > 0.171) according to whether nurses were designated to the non-COVID (29.34 ± 4.89) or COVID ward (29.02 ± 5.43). However, a significant difference was found in emotional intelligence (*t* = 5.22; *p* < 0.001), with the non-COVID ward nurses scoring higher (5.82 ± 0.645) than COVID ward nurses.

While no significant differences were found in resilience according to age (*F* = 0.663; *p* = 0.618), age was found to be significantly related to emotional intelligence (*F* = 6.67; *p* < 0.001), specifically older nurses.

Years of experience revealed no significant relationship with resilience (*F* = 0.947; *p* = 0.418), but it did have a significant relationship with emotional intelligence (*F* = 3.036; *p* = 0.030). Finally, nationality was found to have a significant relationship with resilience (*t* = 6.422; *p* < 0.001) with non-Saudis scoring higher, but not with emotional intelligence (*t* = 0.485; *p* = 0.181).

Table 3 shows the relationship between resilience and emotional intelligence. Resilience was found to have a moderate positive relationship with emotional intelligence (*r* = 0.55; *p* = 0.023). There was a weak positive relationship between resilience and self-emotion (*r* = 0.21; *p* = 0.003) and a very strong relationship between resilience and emotional appraisal (*r* = 0.85; *p* < 0.001). There was no significant relationship between resilience and the regulation of emotions (*r* = 0.69; *p* = 0.265) or the use of it (*r* = 0.65; *p* = 0.295) (Table 3).

## 6. Discussion

This study provides information on the relationships between demographic characteristics, resilience, and emotional intelligence. It also provides an understanding of the association between resilience and emotional intelligence during the COVID-19 pandemic. The results showed that there was no significant difference in resilience according to sex. This is in line with a study by Yu and colleagues [29], who also found that sex was not an influencing factor in resilience. However, this result disagrees with those of a few studies [30,31] that have indicated that women are more resilient than their male counterparts. Sex was significantly related to emotional intelligence in the current study, with the results suggesting that female nurses were more emotionally intelligent than male nurses. Caution should be exercised with this interpretation because of the low number of male participants. It may also be attributed to the fact that nursing was historically founded by women, who are expected to have more emotional intelligence than men. The difference between the emotional intelligence of female and male nurses could be attributed to the social differences between women and men and how they express their emotions. Such a result held true for Pande [32], who also concluded that mean emotional intelligence scores were higher in women. Women are encouraged to be more outspoken, whereas men are trained to suppress their emotions to maintain their patriarchal image [33]. In times of crisis, men tend to talk less, and they consequently receive less help and empathy than women [3]. The sex difference could be because male and female nurses differ in the personality traits that affect how they manage a crisis. Likewise, sex could limit the innate manifestation of resilience in women, while permitting it in men [34]. Additionally, nurturance and interpersonal connections are valued more by females than by males [35].

The nurses’ designations to the COVID or non-COVID ward were not found to make a significant difference to resilience. The results of this study demonstrate that resilience refers to a nurse’s capacity to adjust to workplace challenges and reduce the adverse consequences of job demands. This outcome is consistent with the research of Jackson et al. [36], who concluded that resilience is a nurses’ ability to identify the positive elements and advantages in a crisis event. It is also consistent with the literature review of Grafton et al. [37], who found that resilience was a natural resource that could be enhanced by interventions, such as education and environmental assistance, to mitigate the consequences of workplace challenges. 

A significant relationship was found between the designated area and emotional intelligence in the present study, indicating that nurses in the non-COVID ward were more emotionally intelligent than those in the COVID ward. Although it is assumed that nurses must possess emotional-intelligence abilities, more is expected of those who are exposed to vulnerable situations, as they must make difficult judgments quickly. McDonalds and colleagues [38] suggested coming up with creative yet practical solutions to challenges and working in teams, in which cooperation and solidarity are essential. Personal attributes, such as hope, self-efficacy, and work–life balance, can help build emotional intelligence. This dependence on emotional intelligence may gradually build up nurses’ awareness of their own EI-related behaviors, thus enabling them to engage actively in ensuring the delivery of high-quality interdisciplinary care [39]. Nurses’ effective communication with patients during end-of-life care may preserve a nurturing environment in the hospital, where hospital employees, particularly nurses, demonstrate a natural ability to communicate empathy. It is important to explore the needs of nurses assigned to the COVID ward more deeply to help them be more resilient and maintain their state of emotional intelligence.

No significant differences were found in resilience according to age. This is in line with a study by Gillespie et al. [40], who found that resilience was not associated with age. However, age was significantly related to emotional intelligence, with older nurses being more emotionally intelligent. This suggests that EI is a dynamically evolving process shaped by life experience. This result may be attributed to the fact that older nurses have more experience engaging with people and patients, thus improving their ability to read the emotions of others. According to Kaufman and colleagues [41], lifelong learning and accumulated knowledge can explain the favorable association between age and emotional intelligence. This result is consistent with earlier studies concluding that older adults have significantly greater EI scores than younger adults [42]. This valuable finding can inform nurses and management about the need to improve emotional intelligence by using and practicing it in the workplace across all age groups. 

Years of experience had no significant relationship with resilience in this study. This finding is in line with former studies [43,44] that have concluded that there is no substantial variation in resilience based on years of experience. To promote resilience, a supportive work environment should be provided for nurses. Access to training, professional advancement, choice of hours, peer support, performance feedback, and scheduling flexibility are among the strategies that could be employed. Preventing unprofessional behavior, such as belittling, harsh criticism, or bullying from experienced nurses, may reduce the negative effects on newly graduating nurses, encouraging them to remain in the nursing sector [45]. 

A significant relationship was found between nurses’ years of experience and emotional intelligence. As nurses gain work experience, they are exposed to adversities and learn to develop EI over time. This is consistent with the study of Alshammari et al. [46], who showed that participants gained experience and became more acquainted with their own emotional intelligence. Nurses need training, practice, and experience to build EI, which can help them manage both their own and others’ emotions, facilitate the expression of authentic emotional responses, improve empathy, and increase their capacity to communicate feelings and thoughts without causing conflict [47]. These findings are essential for understanding how nurses deal with uncertainties in their work during the pandemic and training them to be more resilient and emotionally intelligent through mentorship programs.

Nationality was found to have a significant relationship with resilience but not with emotional intelligence, with non-Saudi nurses scoring higher on resilience than Saudi nurses. Ward et al. [48] explained that people can settle in and function physically and psychologically in a new setting through the process of adjusting to a new cultural context, whether out of choice or necessity. According to Lecerof and colleagues [48], due to personal, societal, and economic reasons that could emerge throughout the acculturation process, immigrants may also be more likely than native-born populations to experience distress.

The study findings indicated that resilience had a moderate positive relationship with emotional intelligence. This means that because nurses can recognize, understand, and control their emotions, they can endure difficulties effectively while delivering outstanding patient care. The findings in the literature support the positive relationship between resilience and emotional intelligence [11,12]. According to Holston and Talor [49], emotional intelligence and resilience are personality traits (such as self-awareness, assertiveness, and coping with stress) that are linked to the ability to deal with obstacles. They enable nurses to manage psychological emotions, while allowing them to think more clearly in critical and stressful situations and to make better judgments [50]. This finding offers a unique opportunity for nurses to maintain and continually improve their resilience and emotional intelligence levels through continuous training. 

The current study’s results showed a weak positive relationship between resilience and self-emotion. This corroborates a study by Schneider et al. [51], who found that resilience has a weak positive association with self-emotion and that emotional intelligence helps people cope resiliently with stress. The findings of this study also indicate that there is a very strong relationship between resilience and the emotional appraisal of others. This implies that if nurses are resilient, they are more able to perceive and comprehend the emotions of those around them. Nurses who are resilient intentionally nurture their positive emotionality and optimistic thinking to evoke the positive feelings [52] that result from others’ emotional appraisals. This suggests that nurses can survive and thrive after a life crisis because they have the social and relational capacities to deal with unexpected and unpleasant circumstances. 

There was no significant relationship between resilience and the regulation of or use of emotions among the nurses in this study. This suggests that nurses have a chance to adapt their emotional responses amid adversities. Emotionally resilient nurses are aware of their emotions and of why they need to experience them [53]. Even in the face of a catastrophe, they maintain realistic optimism and are proactive in utilizing internal and external resources [54]. As a result, these nurses can cope with stress and emotions in a healthy and constructive way, which improves the quality of nursing care in terms of clinical performance [55]. This critical finding suggests that policymakers should include resilience and emotional intelligence in the continuous professional development of nurses to train them how to advocate for themselves, how to solve difficulties, and how to seek assistance when necessary. 

## 7. Limitations

This study was subject to limitations that invite future researchers to conduct further studies. The exclusion of nurses working in private and primary healthcare clinics may enhance the representativeness of the sample. Moreover, this study was conducted in only one region of Saudi Arabia. The study could be replicated in a broader context that considers large regions and cities.

## 8. Conclusions

Although nurses’ sex, designated ward, age, and years of experience were found to have no significant relationship with resilience, they did have a significant relationship with emotional intelligence. Resilience was found to have a moderate positive relationship with emotional intelligence, a weak positive relationship with self-emotion, a very strong relationship with emotional appraisal, and no significant relationship with the regulation and use of emotion. Support structures and interventions must be put in place to help every nurse during pandemic outbreaks. Emotional-intelligence training is crucial because it enhances nurses’ individual and social resilience, as well as their professional and personal lives. These findings point to the integration of emotional and intellectual data into therapeutic decision-making and the incorporation of emotional intelligence into clinical practice. A nurse’s resilience can assist them in proactively recognizing or preventing possible difficulties, reducing emotional weariness, promoting work engagement, improving function when faced with workplace challenges, encouraging job resources, and eventually attaining personal and professional progress.

The findings of this study suggest that employers need to recognize nurses’ resilience and their emotional intelligence, not only during the pandemic. This can help nurses proactively identify ways to prevent anxiety, stress, and psychiatric problems. Moreover, there is a need to understand resilience and emotional intelligence to prevent emotional exhaustion, increase work engagement, and improve function when confronted with workplace challenges, ultimately cultivating job resources and attaining personal and professional growth. It is important that hospital authorities prepare their employees to be more emotionally intelligent and resilient by developing educational strategies that enhance professional and personal outcomes.

## Figures and Tables

**Table 1:** Demographic characteristics of staff nurses, *n* = 261.

Demographics	Frequency	Percentage
Age group		
25 or less	28	10.7
26–30	78	29.9
31–35	89	34.1
36 or older	66	25.3
Sex		
Male	121	46.4
Female	140	53.6
Years of experience		
5 years or less	81	31.0
6–10 years	92	35.2
11–15 years	51	19.5
16 years or more	37	14.2
Designated ward		
Non-COVID ward	110	42.1
COVID ward	151	57.9
Nationality		
Saudi	120	45.9
Non-Saudi	141	54.1

**Table 2:** Differences between demographic characteristics, resilience, and emotional intelligence.

Variable	Group	Mean	Std	df	*t*/*F*	*p*-Value
Sex						
Resilience	Male	28.38	4.594	259	*t* = −1.013	0.886
	Female	29.36	5.103			
EI	Male	4.34	2.158	259	*t* = −7.890	0.001
	Female	5.78	0.644			
Designated area						
Resilience	Non-COVID Ward	29.3	4.893	259	*t* = 0.456	0.171
	COVID Ward	29.0	5.433			
EI	Non-COVID Ward	5.82	0.645	259	*t* = 5.224	0.001
	COVID Ward	5.10	1.578			
Age in years						
Resilience	25 and below	29.9	3.540	4, 256	F = 0.663	0.618
	26–30	29.6	4.144			
	31–35	29.3	6.022			
	36 and older	28.4	5.138			
EI	25 and below	4.68	1.942	4, 256	F = 6.674	0.001
	26–30	5.74	1.024			
	31–35	5.62	0.868			
	36 and older	5.82	0.535			
Years of experience						
Resilience	5 years or less	29.54	4.040	3, 257	F = 0.947	0.418
	6–10 years	29.40	5.931			
	11–15 years	28.19	3.538			
	16 years or more	29.67	6.276			
EI	5 years or less	5.36	1.273	3, 257	F = 3.036	0.030
	6–10 years	5.77	0.711			
	11–15 years	5.82	0.599			
	16 years or more	5.48	1.560			
Nationality						
Resilience	Saudi	29.21	4.76	259	*t* = 6.422	0.001
	Non-Saudi	29.52	5.21			
EI	Saudi	5.79	0.692	259	*t* = 0.485	0.181
	Non-Saudi	5.12	1.492			

Std = standard deviation; *t* = *t*-value; *F* = *F*-value; significant at 0.05.

**Table 3:** Relationship between resilience and emotional intelligence.

	Variable	*r*	*P*	Interpretation
Resilience	Total emotional intelligence	0.55	0.023	Moderate positive relationship
	Self-emotional appraisal	0.21	0.003	Weak positive relationship
	Regulation of emotion	0.69	0.265	No significant relationship
	Use of emotion	0.65	0.295	No significant relationship
	Emotional appraisal of others	0.85	0.001	Very strong relationship

Significant at 0.01.

## Data Availability

The data presented in this study are available on request from the corresponding author.

## References

[1] Wu W., Zhang Y., Wang P., Zhang L., Wang G., Lei G., Xiao Q., Cao X., Bian Y., Xie S. (2020). Psychological stress of medical staffs during outbreak of COVID-19 and adjustment strategy. J. Med. Virol..

[2] Henrich N.J., Dodek P.M., Gladstone E., Alden L., Keenan S.P., Reynolds S., Rodney P. (2017). Consequences of Moral Distress in the Intensive Care Unit: A Qualitative Study. Am. J. Crit. Care.

[3] Sun J., Stewart D. (2007). Age and Gender Effects on Resilience in Children and Adolescents. Int. J. Ment. Health Promot..

[4] Alharbi J., Jackson D., Usher K. (2020). The potential for COVID-19 to contribute to compassion fatigue in critical care nurses. J. Clin. Nurs..

[5] Montemurro N. (2020). The emotional impact of COVID-19: From medical staff to common people. Brain Behav. Immun..

[6] Hart P.L., Brannan J.D., De Chesnay M. (2014). Resilience in nurses: An integrative review. J. Nurs. Manag..

[7] Mealer M., Jones J., Moss M. (2012). A qualitative study of resilience and posttraumatic stress disorder in United States ICU nurses. Intensiv. Care Med..

[8] Bar-On R. (2002). Bar-On Emotional Quotient Inventory: Short. Technical Manual.

[9] Fletcher D., Sarkar M. (2016). Mental fortitude training: An evidence-based approach to developing psychological resilience for sustained success. J. Sport Psychol. Action.

[10] Windle G., Bennett K.M., Noyes J. (2011). A methodological review of resilience measurement scales. Health Qual. Life Outcomes.

[11] DePierro J., Lowe S., Katz C. (2020). Lessons learned from 9/11: Mental health perspectives on the COVID-19 pandemic. Psychiatry Res..

[12] Kok N., Hoedemaekers A., Van Der Hoeven H., Zegers M., Van Gurp J. (2020). Recognizing and supporting morally injured ICU professionals during the COVID-19 pandemic. Intensiv. Care Med..

[13] Delgado J., Siow S., de Groot J., McLane B., Hedlin M. (2021). Towards collective moral resilience: The potential of communities of practice during the COVID-19 pandemic and beyond. J. Med. Ethics.

[14] Cooper A.L., Brown J.A., Rees C.S., Leslie G.D. (2020). Nurse resilience: A concept analysis. Int. J. Ment. Health Nurs..

[15] Cam O., Büyükbayram A. (2015). The Results of Nurses’ Increasing Emotional Intelligence and Resilience. J. Psychiatr. Nurs..

[16] Hurley J., Hutchinson M., Kozlowski D., Gadd M., Vorst S. (2020). Emotional intelligence as a mechanism to build resilience and non-technical skills in undergraduate nurses undertaking clinical placement. Int. J. Ment. Health Nurs..

[17] Trigueros R., Padilla A.M., Aguilar-Parra J.M., Rocamora P., Morales-Gázquez M.J., López-Liria R. (2020). The Influence of Emotional Intelligence on Resilience, Test Anxiety, Academic Stress and the Mediterranean Diet. A Study with University Students. Int. J. Environ. Res. Public Health.

[18] Chen S. (2019). Chinese Adolescents’ Emotional Intelligence, Perceived Social Support, and Resilience—The Impact of School Type Selection. Front. Psychol..

[19] Magnano P., Craparo G., Paolillo A. (2016). Resilience and emotional intelligence: Which role in achievement motivation. Int. J. Psychol. Res..

[20] Di Fabio A., Saklofske D.H. (2018). The contributions of personality and emotional intelligence to resiliency. Pers. Individ. Differ..

[21] Bulathwaa A. (2013). Trauma and Coping among University Students: Exploring Emotional Intelligence Application on Coping with Trauma. Arbeit-stitle-Forum für Leipziger Promovierende.

[22] Tu Z.-H., He J.-W., Zhou N. (2020). Sleep quality and mood symptoms in conscripted frontline nurse in Wuhan, China during COVID-19 outbreak: A Cross-Sectional Study. Medicine.

[23] Pasay-An E. (2020). Exploring the vulnerability of frontline nurses to COVID-19 and its impact on perceived stress. J. Taibah Univ. Med Sci..

[24] Campbell-Sills L., Stein M.B. (2007). Psychometric analysis and refinement of the connor–davidson resilience scale (CD-RISC): Validation of a 10-item measure of resilience. J. Trauma. Stress.

[25] Aloba O., Olabisi O., Aloba T. (2016). The 10-Item Connor-Davidson Resilience Scale: Factorial Structure, Reliability, Validity, and Correlates Among Student Nurses in Southwestern Nigeria. J. Am. Psychiatr. Nurses Assoc..

[26] Law K.S., Wong C.-S., Song L.J. (2004). The Construct and Criterion Validity of Emotional Intelligence and Its Potential Utility for Management Studies. J. Appl. Psychol..

[27] Park H.-J., Yu S. (2021). Validity and Reliability of the Korean version of the Wong and Law Emotional Intelligence Scale for Nurses. SAGE Open.

[28] Schober P., Boer C., Schwarte L.A. (2018). Correlation Coefficients: Appropriate Use and Interpretation. Anesth. Analg..

[29] Yu F., Raphael D., Mackay L., Smith M., King A. (2019). Personal and work-related factors associated with nurse resilience: A systematic review. Int. J. Nurs. Stud..

[30] Ren Y., Zhou Y., Wang S., Luo T., Huang M., Zeng Y. (2017). Exploratory study on resilience and its influencing factors among hospital nurses in Guangzhou, China. Int. J. Nurs. Sci..

[31] Netuveli G., Wiggins R.D., Montgomery S.M., Hildon Z., Blane D. (2008). Mental health and resilience at older ages: Bouncing back after adversity in the British Household Panel Survey. J. Epidemiol. Community Health.

[32] Pande H.S. (2010). Evaluating Characteristics and Emotional Intelligence among Workers in Organizations in the State of Rajasthan. Int. Res. J..

[33] Naghavi F., Redzuan M. (2011). The Relationship between Gender and Emotional Intelligence. World Appl. Sci. J..

[34] Shanahan M.J., Hofer S.M. (2005). Social Context in Gene–Environment Interactions: Retrospect and Prospect. J. Gerontol. Ser. B Psychol. Sci. Soc. Sci..

[35] Gunkel M., Lusk E.J., Wolff B., Li F. (2007). Gender-specific Effects at Work: An Empirical Study of Four Countries. Gend. Work. Organ..

[36] Jackson D., Firtko A., Edenborough M. (2007). Personal resilience as a strategy for surviving and thriving in the face of workplace adversity: A literature review. J. Adv. Nurs..

[37] Grafton E., Gillespie B., Henderson S. (2010). Resilience: The Power Within. Oncol. Nurs. Forum.

[38] McDonald G., Jackson D., Wilkes L., Vickers M.H. (2012). A work-based educational intervention to support the development of personal resilience in nurses and midwives. Nurse Educ. Today.

[39] Bailey C., Murphy R., Porock D. (2011). Professional tears: Developing emotional intelligence around death and dying in emergency work. J. Clin. Nurs..

[40] Gillespie B.M., Chaboyer W., Wallis M., Grimbeek P. (2007). Resilience in the operating room: Developing and testing of a resilience model. J. Adv. Nurs..

[41] Kaufman A.S., Johnson C.K., Liu X. (2008). A CHC Theory-Based Analysis of Age Differences on Cognitive Abilities and Academic Skills at Ages 22 to 90 Years. J. Psychoeduc. Assess..

[42] Tsaousis I., Kazi S. (2013). Factorial invariance and latent mean differences of scores on trait emotional intelligence across gender and age. Personal. Individ. Differ..

[43] Pannell L.M., Rowe L., Tully S. (2017). Stress Resiliency Practices in Neonatal Nurses. Adv. Neonatal Care.

[44] Sezgin F. (2012). İlköğretim okulu öğretmenlerinin psikolojik dayanıklılık düzeylerinin incelenmesi. Kastamonu Eğitim Dergisi.

[45] Freeling M., Parker S. (2015). Exploring experienced nurses’ attitudes, views and expectations of new graduate nurses: A critical review. Nurse Educ. Today.

[46] Alshammari F., Pasay-An E., Gonzales F., Torres S. (2020). Emotional intelligence and authentic leadership among Saudi nursing leaders in the Kingdom of Saudi Arabia. J. Prof. Nurs..

[47] Hussien R.M., El Kayal M.M., Shahin M.A.H. (2020). Emotional Intelligence and Uncertainty among Undergraduate Nursing Students during the COVID-19 Pandemic Outbreak: A Comparative Study. Open Nurs. J..

[48] Ward C., Bochner S., Furnham A. (2020). The Psychology of Culture Shock.

[49] Holston E.C., Taylor J.Y. (2016). Emotional Intelligence in Nursing Students. Int. J. Adv. Psychol..

[50] Kelishami F.G., Farahani M.A., Orak R.J., Ameri Z.A., Hashemi S.B., Seyedfatemi N. (2017). Emotional Intelligence in Nursing, Models and Methods of Measurement. Adv. Nurs. Midwifery.

[51] Schneider T.R., Lyons J.B., Khazon S. (2013). Emotional intelligence and resilience. Personal. Individ. Differ..

[52] Kumpfer K.L., Glantz M.D., Johnson J.L. (1999). Resilience and Development: Positive Life Adaptations.

[53] Davenport L. (2017). Emotional Resiliency in the Era of Climate Change: A Clinician’s Guide.

[54] Sisto A., Vicinanza F., Campanozzi L.L., Ricci G., Tartaglini D., Tambone V. (2019). Towards a Transversal Definition of Psychological Resilience: A Literature Review. Medicina.

[55] Jang R.-J., Kang Y.-S., Kim Y.-M. (2016). The Relationships in Emotional Intelligence, Job Satisfaction, and Quality of Nursing Service in Hospital Nurses. J. Korea Acad.-Ind. Coop. Soc..

